# Wild and Cattle Diseases1Read before the Thirty-fifth Annual Meeting of the United States Veterinary Medical Association, September, 1898.

**Published:** 1898-09

**Authors:** H. D. Fennimore

**Affiliations:** Knoxville, Tenn.


### WILD AND CATTLE DISEASES.[1]

By H. D. Fennimore, D.V.S.,
KNOXVILLE, TENN.

For several years past large numbers of cattle have died in East Tennessee of a malady known as "wild" and "cattle-disease." It continued in its deadly course until quite a serious outbreak of it occurred in the State Experiment Station herd, when Dr. Charles W. Dabney, the President of the University, ordered it investigated. It was recognized through the assistance of Dr. Norgaard as the disease described by Bollinger in 1878 under the denomination of "Wild und Rinderseuche."

It is a specific infectious disease that attacks very many species of animals, such as the deer, elk, wild and domestic hog, and cattle. It has also been seen in the horse and dog. In certain sections of Europe, notably the German Empire, it has killed large numbers of wild animals confined in parks, as well as domestic cattle. In this country it has been observed in Virginia, Texas, and now in Tennessee.

The symptoms are loss of appetite, constipation, diminished flow of milk, increase of temperature in the early stages of the disease; after the animal gets very anæmic the temperature drops to about normal. The mucous membranes become very pale, the constipation is followed by diarrhœa, and the feces are frequently stained with blood. The intestines are more or less distended with gas. There is a dropsical swelling hanging from between the lower jaws that preserves the imprint of the finger. This symptom, with the loose, bloody

[1] Read before the Thirty-fifth Annual Meeting of the United States Veterinary Medical Association, September, 1898.

condition of the bowels, is very characteristic, and aids very much in an easy recognition of the disease. In some cases the soft parts of the head, face, neck, shoulders, brisket, and lower part of the legs are the seat of large œdematous swellings. The hair is bristly, and the epidermic desquamation produces an abundant furfur, and in a few cases ulcerations of the skin were noticed.

In the acute form these symptoms may increase rapidly and produce death in a few days or a week, while in a chronic form they may last for months or even a year. From what we can ascertain the disease takes a milder and more chronic form than in the old country, yet at any time it may assume a more virulent character.

Dr. Salmon speaks of it as an infectious ulcerative enteritis complicated with œdematous swellings under the jaws, neck, and brisket, and frequently accompanied by inflammatory lesions of the pectoral organs. He also says it is caused by a specific microorganism that belongs to the swine-plague group, and resembles that bacillus very much.

On post-mortem examination the digestive mucous membrane of the fourth stomach and intestines is inflamed, swollen, and dotted with ulcers. The mucous membrane from the seat of the ulcers in some cases remains attached on one side and hangs as a flap. The ulcers are particularly noticeable at the outlet of the fourth stomach, along the course of the small intestines and at the ilio-cæcal valve. These ulcers are seen in various stages of development, from as large as two inches in length, and smaller ones that are in the process of repair, to cicatricial remains of where they have healed perfectly. Throughout the intestinal tract are found hemorrhages of greater or less extent, with blood-clots and a bloody liquid condition of the contents. Diarrhœa being present in most cases, the lungs are at times filled with gelatinous infiltration. The pleura is inflamed, swollen, and the visceral layer adhering to that of the chest-wall. The outside of the heart shows inflammatory new-growth, the pericardium is inflamed, and it, as well as the chest-cavity, contains a variable amount of dropsical liquid. Croupous lesions are found in the respiratory and intestinal tracts. The lymphatic glands are enlarged and œdematous. The subcutaneous connective tissue over the swollen parts is very much thickened and infiltrated. The abdominal cavity contains more or less liquid, and hemorrhages of the muscles and intestines are very common. All these symptoms and post-mortem lesions were noticed in one animal, but the majority of cases seen were of the intestinal type.

It is not well known how the bacillus enters the system of the affected animals, but it is probable that it may enter through wounds of the skin. It may also be taken in with inspired air and gain entrance through the respiratory mucous membrane. The most probable source of infection of the subjects that came under our notice is through the medium of the food or drinking-water, thus accounting for so many cases affected with lesions of the alimentary canal.

In the absence of inspection laws the meat and milk of the affected animals have been consumed, but there has been no case reported where the disease was communicated to man.

